# Mitochondrial quality control: from molecule to organelle

**DOI:** 10.1007/s00018-021-03775-0

**Published:** 2021-03-29

**Authors:** Alba Roca-Portoles, Stephen W. G. Tait

**Affiliations:** 1 Institute of Cancer Sciences, Cancer Research UK Beatson Institute, University of Glasgow, Switchback Road, Glasgow, G61 1BD UK; 2 Present Address: Centro de Biología Molecular Severo Ochoa (CBMSO), Nicolás Cabrera, 1, 28049 Madrid, Spain

**Keywords:** Mitochondrial dysfunction, UPR^mt^, ISR, Mitochondrial fission, Mitochondrial fusion, Mitophagy, PINK1, Parkin, Mitochondrial diseases

## Abstract

Mitochondria are organelles central to myriad cellular processes. To maintain mitochondrial health, various processes co-operate at both the molecular and organelle level. At the molecular level, mitochondria can sense imbalances in their homeostasis and adapt to these by signaling to the nucleus. This mito-nuclear communication leads to the expression of nuclear stress response genes. Upon external stimuli, mitochondria can also alter their morphology accordingly, by inducing fission or fusion. In an extreme situation, mitochondria are degraded by mitophagy. Adequate function and regulation of these mitochondrial quality control pathways are crucial for cellular homeostasis. As we discuss, alterations in these processes have been linked to several pathologies including neurodegenerative diseases and cancer.

## Introduction

Mitochondria are implicated in an expanding array of biological processes including redox balance, calcium homeostasis, energy production, metabolism and cell death [1, 2]. To maintain function, cells have evolved various processes that sense and respond to defective mitochondrial activity. These mechanisms react differently depending on the nature or intensity of the stress that mitochondria face. For instance, mitochondria can sense internal stresses such as misfolded proteins, mitochondrial DNA (mtDNA) mutations, metabolic or oxidative stress [3]. Mitochondria can adapt to these by retrograde signaling to the nucleus leading to the transcriptional upregulation of stress response proteins [4, 5]. Besides these stresses, mitochondria also face external ones such as mechanical stress, infection and environmental stress (e.g., hypoxia) [3]. Hence, when cells are challenged, mitochondria can maintain function through a second line of defense, by altering their mitochondrial dynamics. However, in the face of persistent damage, another homeostatic mechanism is to remove damaged mitochondria through a process called mitophagy [3, 6, 7].

Alterations in mitochondrial quality control responses have been described in several mitochondrial diseases such as neurodegenerative diseases (Parkinson’s, Alzheimer’s and Huntington’s disease) [8–11], cardiomyopathies [12], ocular diseases [13, 14] and cancer [15–17], highlighting the importance of an adequate balance of these pathways for maintaining homeostasis.

In this review, we will discuss key mechanisms of mitochondrial quality control pathways. Due to the complexity and diversity of these pathways we have divided them into two broad areas: regulation at the molecular level (focusing on mitochondrial-to-nuclear communication) and regulation at the organelle level (including mitochondrial dynamics and mitophagy). Our discussion is not intended to be exhaustive, for instance, for in-depth discussion of the cellular response to ROS, NAD + (nicotinamide adenine dinucleotide) and calcium signaling, the reader is referred to recent, comprehensive reviews [18–20].

### Regulation of mitochondrial quality control at the molecular level

**Fig. 1:**
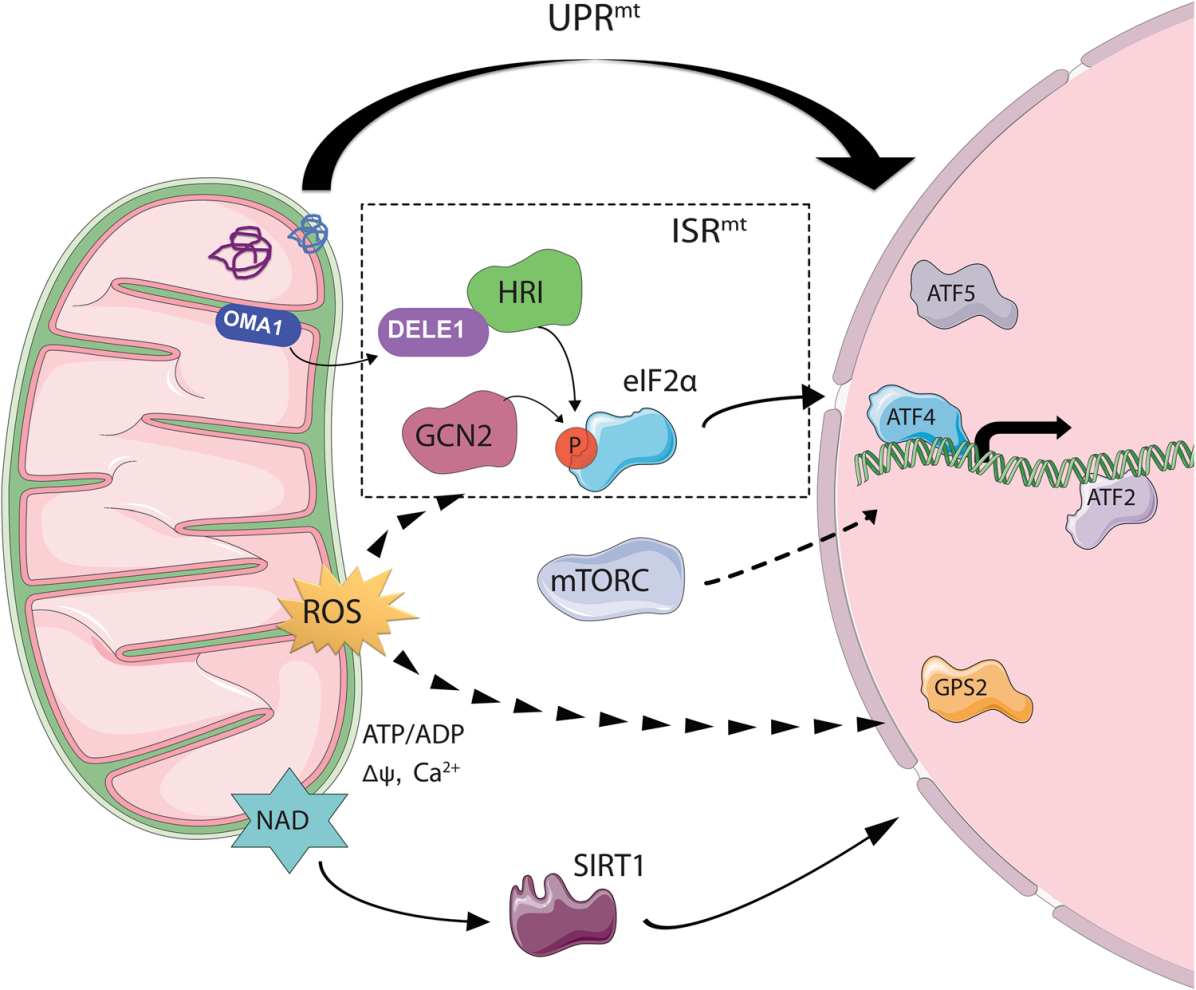
Mitochondrial retrograde signaling. The mitochondrial integrated stress response (ISR^mt^) is regulated by phosphorylation of the elongation transcription factor eIF2α, which enhances the translation of ATF4. The kinases HRI and GCN2 phosphorylate eIF2α (P-eIF2α) following a mitochondrial stress. In addition, the ISR is also part of the mitochondrial unfolded protein response (UPR^mt^), activated by misfolded mitochondrial proteins. Moreover, other mechanisms of mito-nuclear communication (mTORC, SIRT1, GPS2) are activated following changes in mitochondrial ROS, NAD, ATP/ADP ratio, calcium (Ca^2+^) or membrane potential (Δψ). Once the transcription factors ATF4, ATF5, ATF2 and/or GPS2 are in the nucleus, they regulate the expression of nuclear stress-response genes

Mitochondria are organelles of bacterial ancestry. Thus, mitochondria have their own genome (mtDNA) in the matrix, which is surrounded by the inner (IMM) and outer mitochondrial membrane (OMM). While mitochondria have their own genome, the vast majority of mitochondrial proteins are encoded in the nucleus [21] and therefore require import to maintain mitochondrial function [22]. Additionally, mitochondria also need to cope with oxidative stress. Therefore, to ensure homeostasis, mitochondria have developed several mechanisms to sense and respond to stress via nuclear communication. This mito-nuclear communication—also termed mitochondrial retrograde signaling or mitohormesis—is an evolutionary conserved process. It can be triggered by several stressors such as misfolded proteins, inhibition of the ETC, mitochondrial depolarization, nutrient deprivation and/or redox imbalances [23–27]. Hence, different pathways are involved in regulating mito-nuclear communication (Fig. 1).

#### Mitochondrial retrograde response

One of the first descriptions of mitochondrial retrograde signaling was made in the yeast *Saccromyces cerevisiae,* where the genes *RTG1* and *RTG2* were found to regulate the expression of the nuclear gene citrate synthase (*CIT2*) in response to alteration of mitochondrial function [28]. Stemming from this finding, Jia et al. described that the transcription factor involved in the regulation of *CIT2* expression in yeast is a heterodimeric complex of Rtg1p and Rtg3p proteins (Retrograde regulation protein 1 and 3) [29]. Recently, it has been shown that inhibition of protein import into mitochondria and consequently cytoplasmic accumulation of misfolded mitochondrial proteins caused global changes at the transcriptome and proteome levels [30]. This led to an increased expression of proteasome subunit and cytosolic chaperones and a decrease in oxidative phosphorylation (OXPHOS) proteins. However, these changes were regulated by different transcription factors; Hsf1 and Rpn4 were involved in the upregulation of the proteasome and chaperones proteins; whilst the inactivation of the HAP complex led to a downregulation of OXPHOS proteins [30]. This study shows that there are multiple pathways that regulate mito-nuclear communication. Indeed, several mitochondrial signaling pathways that respond to inhibition of protein import have been recently described in yeast such as the unfolded protein response activated by mistargeting of proteins (UPR^am^) [31], mitochondrial precursor over-accumulation stress (mPOS) [32], mitochondrial compromised protein import response (MitoCPR) [33] and mitochondrial protein translocation-associated degradation (mitoTAD) [34], reviewed in [35].

Mitochondrial retrograde signaling has also been described in the nematode *Caenorhabditis elegans*, where mitochondria can sense and respond to unfolded proteins by activating the mitochondrial unfolded protein response (UPR^mt^), and thereby regulating the expression of mitochondrial chaperone genes, amongst others [36]. Mechanistically, Haynes and colleagues found that upon a proteotoxic stress, the mitochondrial matrix protease CLPP and the homeodomain-containing transcription factor DVE-1 in complex with the small ubiquitin-like protein UBL-5, are required for UPR^mt^ [37]. Additionally, they found that both CLPP and the mitochondrial peptide transporter, HAF-1, are required for activation of the transcription factor ZC376.7, which binds to and activates the expression of nuclear genes involved in the UPR^mt^ [38]. The transcription factor ZC376.7 (mammalian homolog of ATF5), was later re-named as ATFS-1 (Activating Transcription Factor associated with Stress-1). Thus, Nargund et al. showed that in *C. elegans*, ATFS-1 is imported into mitochondria and degraded under basal conditions, but following a mitochondrial stress, ATFS-1 accumulates in the cytosol, promoting its nuclear transport [39]. In the nucleus, ATFS-1 regulates the expression of several nuclear genes involved in immunity, mitochondrial chaperones and metabolism, amongst others [40, 41].

Besides these, other complementary mitochondrial retrograde signaling pathways have been observed in worms in response to mitochondrial dysfunction and oxidative stress (ROS). For instance, the translation initiator factor eIF2α (Eukaryotic Translation Initiation Factor 2A) is phosphorylated by the kinase CGN2 (general control non-derepressible-2) and modulates cytosolic protein synthesis [42]. On the other hand, the mitochondrial protein monooxygenase CLK-1 can translocate to the nucleus where it regulates ROS metabolism and proteostasis, by suppressing a subset of UPR^mt^ genes [43]. Additionally, Herholz et al. found that Krüppel-like factor 1 (KLF-1) is the key transcription factor that regulates the expression of genes involved in xenobiotic detoxification processes [44]. This argues that distinct mechanisms are also involved in mito-nuclear communication in nematodes.

Furthermore, mitochondrial retrograde signaling has also been described in the fruit fly *Drosophila melanogaster* [45, 46] and in mammals. In mammals, there is evidence that treatments that alter mitochondria health, such as loss of membrane potential, unfolded proteins, inhibition of protein translation or mutations in mitochondrial DNA, signal to the nucleus in order to regulate the expression of nuclear genes and adapt towards adverse conditions [23–27]. Thus, indicating—as a generalized process—retrograde signaling is evolutionary conserved.

One of the mitochondrial retrograde signaling pathways in mammals is the UPR^mt^, which, as in other species, is activated upon the accumulation of misfolded mitochondrial proteins. A similar mechanism has been previously documented in other mammalian organelles, notably the endoplasmic reticulum (ER), where three different pathways (ATF6, PERK, IRE1α) are known to regulate the endoplasmic reticulum unfolded protein response (UPR^er^) [47]. Upon the discovery of UPR^mt^ in other organisms, there has been intensive research trying to identify transcription factors involved in the mammalian UPR^mt^. One possibility is the protein ATF5 (Activated Transcription Factor 5), that appears to be activated similarly to ATFS-1 [48], meanwhile others have reported ATF4 (Activated Transcription Factor 4) as the transcription factor activated upon mitochondrial stress [5]. In addition, there is also evidence in both nematodes and mammalian cells, that NAD + can activate the UPR^mt^ through activation of the Sirtuin pathway (specifically SIRT1) [49].

Another mechanism involved in the mitochondrial retrograde signaling is the integrated stress response (ISR). This pathway is activated upon several stresses such as proteostasis defects, nutrient deprivation, redox imbalances and viral infection, amongst others. However, all of them converge in the phosphorylation of the translation initiator factor eIF2α [50]. Various kinases that phosphorylate eIF2α have been identified: the double-stranded RNA-activated protein kinase (PKR) [51, 52], the general control non-derepressible-2 (GCN2) [53], the endoplasmic reticulum (ER) resident kinase (PERK) [54] and heme-regulated inhibitor (HRI) [55]. Upon phosphorylation of eIF2α (P-eIF2α), the activity of eIF2B (eIF2′s guanine nucleotide exchange factor) is blocked, therefore inhibiting protein synthesis [56]. Nevertheless, P-eIF2α can enhance the translation of the transcription factor ATF4 [57]. ATF4 was first described as a transcription factor involved in the UPR^er^ upon activation by PERK [57]. Nevertheless, different studies have shown that eIF2α and ATF4 can be activated not only upon ER stress but also upon mitochondrial stress engaged by other signaling pathways [5, 42]. Two recent papers have described a mechanism of activation of ATF4 following a mitochondrial stress [58, 59]. Employing a genome-wide CRISPR screen, they found that OMA1 (a mitochondrial stress protease located on the inner mitochondrial membrane) is activated upon mitochondrial stress and cleaves DELE1 (another inner mitochondrial membrane protein). Cleavage of DELE1 leads to its accumulation in the cytosol and therefore activation of HRI, one of the kinases that phosphorylates eIF2α [58, 59].

Interestingly, the expression of not only ATF4 but also ATF5 is enhanced by eIF2α [60], thereby indicating that activation of the ISR is required for activation of UPR^mt^ in mammals. In addition, in muscle, mTORC1 has been shown to regulate the one carbon metabolism and metabolic cytokines (FGF21) via ATF4 signaling [61], indicating a role for mTORC1 also in the ISR. Furthermore, Cardamone and colleagues described that the protein GPS2, involved in inflammation and lipid homeostasis, regulate the transcription of several ISR regulated genes [62]. They found that this protein resides in the mitochondria and, upon mitochondrial depolarization induced by FCCP treatment (a mitochondrial uncoupler of oxidative phosphorylation that disrupts ATP synthesis); GPS2 translocates to the nucleus, upregulating the expression of nuclear genes involved in the tricarboxylic acid (TCA) cycle, the electron transport chain (ETC), lipid synthesis and interleukin signaling pathways [62].

Although less well-described, apart from these, other mechanisms of mitochondrial retrograde signaling have also been reported in mammals. For instance, in myocytes, different mitochondrial stressors (decrease in mitochondrial membrane potential, inhibition of electron transport chain, reduced mtDNA or increased intracellular calcium levels) caused alterations in the expression of nuclear genes. These nuclear genes were regulated by the transcription factors NFAT (cytosolic counterpart of activated T-cell-specific nuclear factor) and ATF2 (Activated transcription factor 2) [27].

In vivo studies have confirmed the existence of several mitochondrial stress pathways in mammals [63–65]. Thus, Gomes et al. showed that when nicotinamide mononucleotide adenylyltransferase (NMNAT1) was decreased in the nucleus, it caused a decrease in expression of mitochondrial OXPHOS genes, mtDNA and ATP levels, by activating SIRT1 [65]. Moreover, mice that lack the expression of the mitochondrial aspartyl-tRNA synthetase, DARS2 (DARS2-deficient mice) display deregulated mitochondrial protein synthesis and thereby activation of the UPR^mt^ [64]. However, although these mice demonstrated alterations in the respiratory chain, activation of the mitochondrial stress response was independent of the respiratory defects observed [64]. Nevertheless, deletion of CLPP in DARS2-deficient mice decreased the observed respiratory defects but did not rescue the UPR^mt^ signaling, indicating a role of CLPP in OXPHOS regulation [63]. In addition, unlike in the nematode *C. elegans* where CLPP has a central role in regulating UPR^mt^ [37], this study showed no role for CLPP in UPR^mt^ regulation in mammals [63]. Hence, these data argue against different mitochondrial stress pathways involved in mito-nuclear communication in vivo that act cooperatively to maintain homeostasis.

#### Mitochondrial retrograde signaling in health and disease

Since the decline of mitochondria function is a hallmark of aging, neurogenerative disease and cancer [66], understanding how those pathways work is important for human health. In fact, several studies have demonstrated the importance of the UPR^mt^ for development and longevity [42, 67]. Thus, mutations or deletions in genes involved in the ETC [68] and increased levels of NAD + [49], activated the UPR^mt^ and extended lifespan. How mito-communication regulates lifespan remains unclear. Gomes et al. demonstrated that SIRT1/HIF1α pathway contributes to aging-associated mitochondrial dysfunction in mice and that this phenotype could be reversed by increasing NAD + levels [65]. This indicates that increasing the levels of NAD + could be beneficial and a possible treatment option for patients with mitochondrial diseases. Similarly, another strategy for treating these patients may be using PARP-1 inhibitors as PARP-1 (poly(ADP-ribose) polymerase-1) is a NAD + consuming enzyme. Indeed, treatment with PARP-1 inhibitors has been demonstrated to restore mitochondrial function [69]. On the other hand, others have suggested that activation of UPR^mt^ extends longevity due to the induction of changes in chromatin structure through histone modifications [70]. In addition, a role for ROS in longevity has also been proposed [43, 46, 71], although others have shown that in mice, accumulation of mtDNA mutations that are associated with an aging phenotype and reduced lifespan, did not affect ROS levels [72]. Recently, a study has found that KLF-1 regulates longevity independently of the UPR^mt^ but dependently on redox signaling [44]. Importantly, that extended longevity was due to the expression of xenobiotic detoxification genes such as cytochrome P450 oxidases (CYPs) [44], proposing CYPs as the key effectors of lifespan extension. However, whether or not the association between mitochondrial dysfunction and longevity is due to ROS, epigenetics or any other mechanisms remains unclear.

Alteration of mitochondrial stress-signaling pathways has also been dysregulated in cancer. For instance, in gastric cancer, cisplatin resistance was found to be due to upregulation of the cystine/glutamate antiporter (xCT) via ROS/GCN2-eIF2α-ATF4 pathway [73]. In addition, other studies have shown that tumours upregulate the ISR as a means of chemoresistance. BRAF-mutated melanoma cells, acquire resistance to vemurafenib by GCN2/ATF4 activation [74] and overexpression of ATF5 conferred radioresistance in lung cancer cells [75] and in malignant glioma cells, ATF5 promoted survival through transcription of anti-apoptotic MCL-1 [76]. Although the involvement of mitochondria was not shown directly in these studies, they offer proof of principle that ISR can induce drug resistance. Recently, ATF4 has also been shown to be oncogenic; in *Drosophila*, activation of ATF4 induced a Warburg-like phenotype and consequently tumorigenic growth [77] and in MYC-driven tumours, deletion of ATF4 prolonged survival [17], indicating that ATF4 is necessary for tumorigenesis. Collectively, although several advances have been made over the recent years in understanding the mitochondrial retrograde response in several organisms, much remains to be elucidated.

### Regulation of mitochondrial quality control at the organelle level

As dynamic organelles, mitochondria can also alter their morphology by undergoing mitochondrial fission or fusion and thereby adapt toward stresses and fulfill the cellular needs. However, if the stress is severe or prolonged, cells can eliminate mitochondria through a process called mitophagy. Both mitochondrial dynamics and mitophagy, have demonstrated their importance for homeostasis as alterations in genes involved in these processes have been found in several diseases [10, 13, 14, 78].

#### Mitochondrial dynamics

**Fig. 2:**
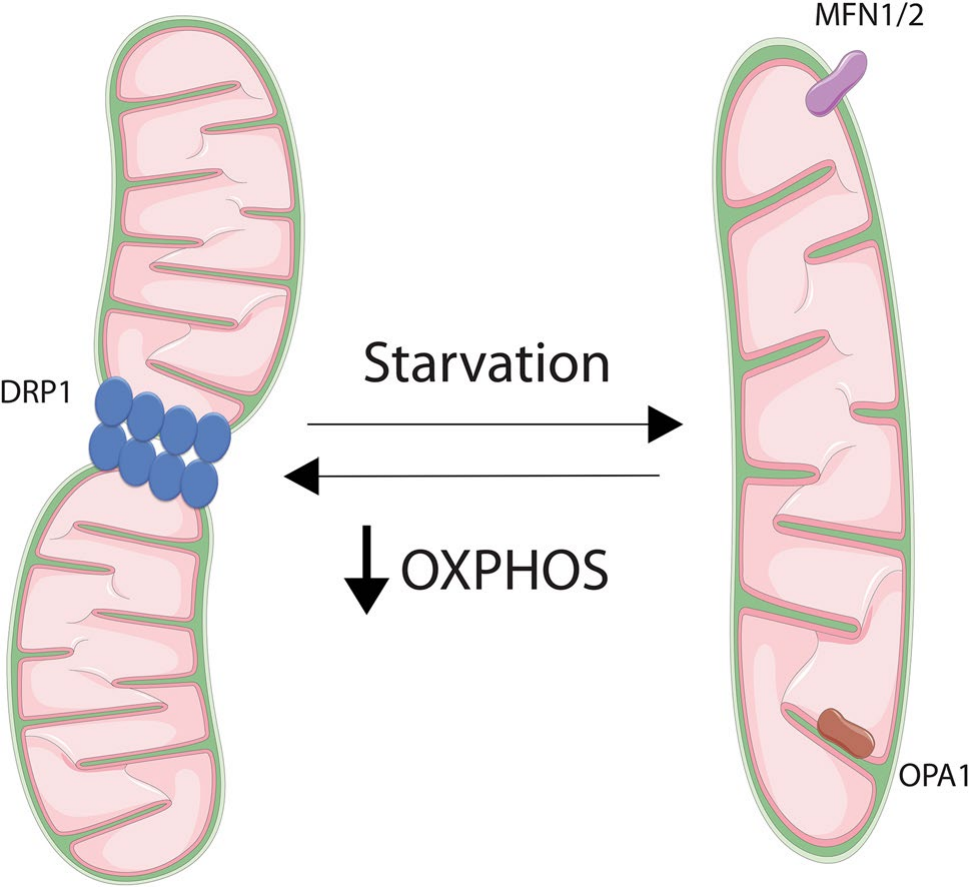
Mitochondrial dynamics. Mitochondria are dynamic organelles that adapt their network according to cellular requirements. For instance, a decrease of respiration leads to mitochondrial fission and starvation induces fusion. Both processes are regulated by different proteins; dynamin-related protein 1 (DRP1) is involved in fission, meanwhile Mitofusin 1 and Mitofusin 2 (MFN1/2) and optic atrophy 1 (OPA1) allows fusion. OXPHOS = oxidative phosphorylation

Mitochondria are dynamic organelles that adapt their network according to cellular requirements. These changes are determined by rates of fission and fusion, the regulation of which varies with regards to the severity of the stresses that they face. Upon a high or prolonged stress, mitochondria undergo fission, meanwhile, upon a mild or low stress, mitochondria favour fusion [79]. Furthermore, studies have shown that mitochondria can also alter their morphology depending on the nature of stress; starvation induces fusion, meanwhile, low glucose and ETC inhibition can induce fission [80–83] (Fig. 2).

An appropriate balance in mitochondrial dynamics is important for mitochondrial health as each of them is implicated in different processes. Fusion is involved in the maintenance of mtDNA integrity, mitochondrial respiration, mitochondrial membrane potential, apoptosis and calcium signalling [84–89]. Fission has been shown to be important for preventing oxidative damage and to enable degradation of damaged mitochondria [6, 87, 90]. Additionally, mitochondrial dynamics have also been implicated in life span regulation [91].

##### Mitochondrial fusion

Mitochondrial fusion is regulated at both mitochondrial membranes; the outer membrane (OMM) and the inner membrane (IMM). Mitofusin 1 (MFN1) and Mitofusin 2 (MFN2) are GTPases required to allow fusion of the OM [92, 93] by forming homodimers or heterodimers [94]. This process is essential for development because combined knockout of MFN1 and MFN2 leads to embryonic lethality. Single knockouts of MFN1 or MFN2 are viable, however, deletion of each one alone promotes mitochondrial fragmentation, demonstrating their role in mitochondrial fusion [94]. In addition, mutations in MFN2 have been found in axonal Charcot-Marie-Tooth disease type 2A [9] and mice with the mutation R95Q in MFN2 developed this pathology [78]. MFN2 deficient mice showed cerebellar defects; dysfunctional ETC and loss of mtDNA nucleoids in Purkinje cells due to dysfunctional mitochondria (fusion-deficient cells) [95]. In another study, MFN2 mutant mice demonstrated a decrease in locomotive activity and a lack of MFN2 in dopaminergic neurons displayed fragmented mitochondria and decreased mitochondrial motility [96]. These data emphasise the importance of this protein and mitochondrial fusion for neurological development.

The protein optic atrophy 1 (OPA1) mediates fusion of the mitochondrial inner membrane [97, 98]. OPA1 has multiple isoforms due to splicing or variants generated via proteolytic cleavage. The long form of OPA1 (L-OPA1) is anchored in the inner membrane and it promotes mitochondrial fusion, whereas the short form of OPA1 (S-OPA1) is soluble. Mitochondrial fusion depends on the presence of both isoforms [99], by forming oligomeric complexes that maintain the cristae structure [100, 101]. Cleavage of L-OPA1 into S-OPA1 is mediated by the AAA-proteases OMA1 [102, 103] and YME1L [99, 104, 105]. Stresses that activate OMA1 or YME1L, for instance loss of mitochondrial potential or OXPHOS activity, lead to the proteolytic process of L-OPA1 into S-OPA1, liberating this protein from the IMM and therefore promoting fission [99, 103, 106, 107]. Thus, regulation of OPA1 proteolysis is a key process for mitochondrial network maintenance and function. Accordingly, mutations in this gene cause dominant optic atrophy (ADOA) [13, 14] and heterozygous mutations have been also associated with Behr syndrome [108].

##### Mitochondrial fission

The main proteins involved in mitochondrial fission are DRP1 (dynamin-related protein 1) [109], mitochondrial fission factor (MFF) [110] and mitochondrial fission protein 1 (FIS1) [111], amongst others [112]. DRP1 is a GTPase that resides in the cytosol. Upon specific stimulus DRP1 is recruited onto the OMM by receptors such as MFF and FIS1 [110]. However, there are differences between the yeast FIS1 and the human Fis1 (hFis1) in their activity. A recent study has shown that hFis1 induces fission independently of DRP1, instead, hFis1 negatively regulates fusion by binding to OPA1, MFN1 and MFN2 and blocking its activity [113]. Nevertheless, once DRP1 gets onto the OMM it multimerizes and forms rings on the mitochondrial outer membrane [109]. Once there, by hydrolysing GTP, the complex changes conformation and allows the constriction of the membrane inducing its division [114]. DRP1 activity is not only regulated by its translocation, but also by post-translational modifications including phosphorylation, ubiquitylation, and SUMOylation. Phosphorylation of S616 allows DRP1 activity [115], meanwhile, phosphorylation of S637 and S656 inhibits it [116, 117]. However, several kinases have been involved in the phosphorylation of its residues. For instance, ERK1/2 [15, 118] and mitotic kinase cyclin B-CDK1 (cyclin-dependent kinase 1 [115] have been described to phosphorylate DRP1 in S616 [119]. On the other hand, S656 is phosphorylated by cyclic AMP‐dependent protein kinase, PKA, and dephosphorylated by calcineurin [117].

DRP1 is essential for development; whole-body deletion of DRP1 is embryonic lethal [120, 121], showing the relevance of this protein for mitochondrial homeostasis. However, unlike MFN1/2 or OPA1, no autosomal diseases have been linked to mutations in DRP1, although alterations in mitochondrial fission have been related to neurodegenerative diseases [8]. Moreover, in different tumour models, such as pancreas [16], melanoma [118], lung cancer [122] and ovarian cancer [123], DRP1 and mitochondrial fission have shown to be important for tumour growth [15]. In addition, tissues from Alzheimer’s disease (AD) [124] and Huntington's disease (HD) [125] patients demonstrated increased expression of DRP1 and FIS1, and decreased expression of MFN1, MFN2 and OPA1, indicating that an impairment on mitochondrial dynamics are associated with AD and HD. Thus, an adequate function of mitochondrial fusion and fission is important for homeostasis and alterations in these processes are associated with several pathologies, such as cancer, neurodegenerative, neuroinflammatory and autoimmune disease [126].

#### Mitophagy

**Fig. 3:**
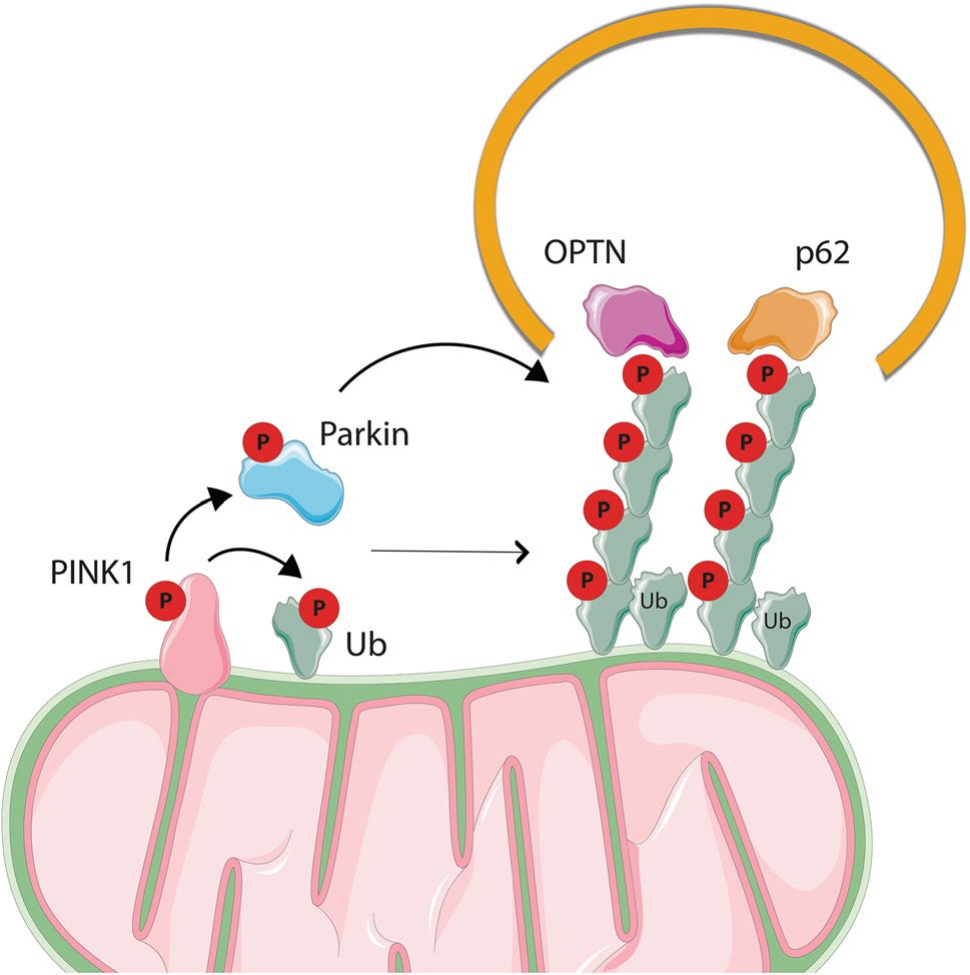
Mitophagy. Mitophagy allows the removal of damaged mitochondria by the PINK/Parkin pathway. When mitochondria are damaged, PINK1 is accumulated on the outer mitochondrial membrane (OMM) where it gets activated and phosphorylates and therefore activates Ubiquitin (Ub) and Parkin. Parkin ubiquitinates different OMM proteins in a feed-forward mechanism, leading to a general mitochondrial ubiquitination. That ubiquitination recruits autophagy receptor proteins (OPTN, p62), and, in turn, autophagosomes into the mitochondria, leading to the induction of autophagy

Autophagy is a regulated process through which cells can degrade unnecessary or non-functional cytosolic components through their engulfment in a double membrane vesicle, called autophagosome, and their delivery into the lysosomes. There are different types of autophagy such as macroautophagy, microautophagy, and chaperone-mediated autophagy (Reviewed in [127, 128]). The selective removal of mitochondria by autophagy, namely mitophagy, helps maintain adequate cellular homeostasis through the elimination of damaged mitochondria [7, 129] (Fig. 3). How was mitophagy discovered? Dysfunctional mitochondria were first observed inside vacuoles in yeast [130]. Later, the term mitophagy was coined by Lemaster’s group after finding mitochondria inside of lysosomes in rat hepatocytes upon nutrient deprivation [131]. These observations raised several questions—how do cells sense damaged mitochondria and how is mitophagy regulated? There are many mechanisms of mitophagy described [132–136]. For instance, the protein NIX has been shown to be involved in the removal of non-damaged mitochondria during erythropoiesis [137, 138]. Here we will focus on the PINK1/Parkin pathway, as the mechanism is better described and this pathway is involved in regulating mitochondrial stress response.

##### PINK1/Parkin pathway

The importance of PINK1 (PTEN-induced putative kinase 1) and Parkin for mitochondrial homeostasis is supported by the accumulation of dysfunctional mitochondria in Parkinson’s disease (PD) models and patients [10, 139]. In fact, both of these proteins have been found mutated in this disease; PINK1 mutation causes Hereditary Early-Onset Parkinson [10] meanwhile mutations in Parkin occurs in Autosomal recessive juvenile Parkinsonism (AR-JP) [11]. In *Drosophila*, studies have shown that this pathway is needed for maintaining adequate mitochondria function in muscle and neurons, and loss of function mutations of both proteins mimic the characteristic of PD [140]. Furthermore, in mice, deletion of Parkin leads to loss of dopaminergic neurons (DA), which is a hallmark of PD [141].

Under normal conditions, PINK1 is partially imported into mitochondria, where it is cleaved by the IMM protease PARL. This cleavage enables PINK1 ubiquitination and proteasome-dependent degradation in the cytosol [142]. Therefore, in healthy mitochondria, the levels of PINK1 are low in cells. Upon stresses such as mitochondrial depolarization [143], impairment of protein import [144], and others [145–147], PINK1 import into mitochondria is blocked and as a result, it accumulates on the OMM [148]. Once there, it binds the mitochondrial TOM complex [149, 150] and it is activated by auto-phosphorylation. Its activation leads to a signaling cascade by recruiting and activating other proteins like Parkin [143, 151] and Ubiquitin (Ub) [152]. Parkin is an E3 ubiquitin ligase that is activated upon PINK1 phosphorylation in its UBL domain [153, 154] and binding of phospho-Ub [152, 155–158]. Once activated, Parkin ubiquitinates different OMM proteins in a feed-forward mechanism, leading to a general mitochondrial ubiquitination. Then, receptor proteins on the mitochondria recruit the autophagic machinery which results in the engulfment of mitochondria by autophagosomes. Different autophagy receptors have been shown to play a role in mitophagy such as NDP52, optineurin (OPTN) and p62 [159, 160].

In vivo, the role of PINK1 and Parkin in mitochondrial homeostasis (mitophagy) is unclear. On the one hand, using a mouse model that lacks the proofreading function of the mitochondrial DNA polymerase g (POLG) (called Mutator mice), Pickrell and colleagues demonstrated that Parkin protects dopaminergic neurons from mitochondrial dysfunction. They show that Parkin loss in Mutator mice led to DA neuron loss and decrease in OXPHOS activity [161], thereby suggesting that Parkin has a role in maintaining mitochondrial homeostasis in vivo, or at least in DA neurons. On the other hand, mice that lack Parkin present normal cardiac function and their mitochondrial activity was unaltered. However, when those mice are challenged with a mitochondrial stress such as myocardial infarction or hypoxia, Parkin played a critical role in removing damaged mitochondria [162]. In line with these findings, another study has corroborated that in heart, the expression of Parkin is dispensable for mitophagy under basal conditions [163]. However, it contributes to mitochondrial removal in cardiomyopathies that present defects in mitochondrial fission [163], thus indicating a role of Parkin in regulating stress-induced mitophagy. In fact, studies have also shown that mice with PINK1 loss display normal basal mitophagy [135] and research in *Drosophila* has corroborated that under basal mitophagy, PINK1 and Parkin are dispensable [164]. Therefore, these studies together will indicate that the PINK1/Parkin pathway may not be implicated in regulating mitophagy under physiological conditions and/or that there are other additional pathways that regulate mitophagy [135]. In addition, although Parkin deficient mice demonstrated no role of Parkin for basal mitophagy, the mitochondria of those mice, although functional, were smaller [162], which may indicate that Parkin has additional roles independently of mitophagy. In fact, Parkin has also been involved in regulating bioenergetics [129], necroptosis [165], mitochondrial protein import [166], mitochondrial biogenesis [167] and inflammation [168].

Another question that remains controversial is whether PD is due to PINK1 or Parkin deficiency or due to a general mitochondrial dysfunction. The aforementioned study demonstrates a role of Parkin and PD [161]. However, pathogenic mutations of PD (T415N and G430D) that abolish the E3 activity of Parkin, altered its mitochondrial localization, whereas other pathological mutations (D280N or G328E) that do not alter its E3 activity did not impact Parkin recruitment to mitochondria [169]. In addition, mutations in PINK1 and Parkin are not exclusive for this pathology as other alterations such as mtDNA mutations [170, 171] and LRRK2 mutations [172, 173] have been described. Moreover, in some PD patients, impairment of MIRO1 degradation, an OMM protein that anchors mitochondria to microtubule motors, has also been observed [174]. Finally, another challenge of studying mitophagy is finding a physiological method of triggering it. Recently, Kovalchuke and colleagues have demonstrated that other more physiological oxidative stressors like L-DOPA, lead to Parkin degradation. Although the exact mechanism of how Parkin is degraded following L-DOPA remains unclear, they show that PINK1 and phospho-Ub are involved in this pathway [175], demonstrating a similar mechanism to other mitochondrial stressors such as CCCP (mitochondrial uncoupler) [156]. This study suggests that oxidative stress or phospho-Ub may contribute to the loss of Parkin in PD. Moreover, that finding opens a new strategy to treat those patients by blocking the association of Parkin with phospho-Ub. Another possible approach for PD treatment comes from the finding that PINK1 is cleaved by OMA1 in depolarised mitochondria. This indicates that inhibition of OMA1 could be used to increase the levels of mitophagy in PD patients [150].

To conclude, mitochondrial damage and defects in mitophagy have been observed in several neurodegenerative diseases, such as Parkinson’s (PD) [176] Alzheimer’s (AD) and Huntington’s disease (HD) [177]. Moreover, mitophagy impairment has also been associated with myopathies, metabolic disorders, inflammation and cancer [178]. Therefore, it is important to understand the mechanisms behind it and its role in the diseases, as it will help to develop new approaches for improved treatments.

## Conclusion

Although the UPR^mt^ is mainly caused by protein misfolding [26], other stresses have also been found to activate the UPR^mt^ [179]. In addition, several conditions that were found to activate the ISR such as alteration of mitochondrial membrane potential, mitochondrial respiration, block in mitochondrial translation or in mitochondrial protein import [5], also alter mitochondrial dynamics [80, 180] and/or activated mitophagy [143, 144]. It is likely that activation of those pathways depends on the severity or duration of the stress. However, even though a threshold may exist, a connection between UPR^mt^ and mitophagy has been reported [144]. Similarly, studies have also demonstrated a relation between mitochondrial dynamics and PINK or mitophagy [181, 182]. Therefore, these studies, amongst others, suggest a connection between the mitochondrial quality pathways, which requires further investigation.

As discussed, imbalances in quality control pathways have been associated with multiple pathologies. For instance, aberrations in mitochondrial dynamics have been found in cancer patients [15, 16], neurodegenerative diseases [8, 9] and ocular diseases [13, 14]. In addition, mutations in mitophagy-related genes have also been reported in neurodegenerative pathological conditions [10, 11]. All these patients have alterations in mitochondrial morphology and function. However, these patients also present mutations in other genes [183], thereby is still unclear whether the development of the diseases is due to alterations in mitophagy itself or rather a general mitochondrial dysfunction. Alterations in the expression of proteins involved in mitochondrial retrograde signaling (ISR) have also been linked to resistance and tumour growth [17, 73, 74, 77]. Hence, even though in the recent years new findings have given some clarity in understanding the mitochondrial quality control pathways, some mechanisms underlying mitochondrial homeostasis remain still unclear. Therefore, investigation of those pathways should provide knowledge for understanding, and potentially treating, mitochondrial pathologies.
